# TD-LSTM: Temporal Dependence-Based LSTM Networks for Marine Temperature Prediction

**DOI:** 10.3390/s18113797

**Published:** 2018-11-06

**Authors:** Jun Liu, Tong Zhang, Guangjie Han, Yu Gou

**Affiliations:** 1 College of Computer Science and Technology, Jilin University, Changchun 130012, China; liujun1509@jlu.edu.cn (J.L.); tongzhang18@mails.jlu.edu.cn (T.Z.); 2 State Key Laboratory of Robotics, Shenyang Institute of Automation, Chinese Academy of Sciences, Shenyang 110016, China; 3 Key Laboratory for Ubiquitous Network and Service Software of Liaoning Province, School of Software, Dalian University of Technology, Dalian 116024, China; hanguangjie@gmail.com

**Keywords:** long short-term memory (LSTM), temporal dependence, sea surface temperature (SST), prediction

## Abstract

Changes in ocean temperature over time have important implications for marine ecosystems and global climate change. Marine temperature changes with time and has the features of closeness, period, and trend. This paper analyzes the temporal dependence of marine temperature variation at multiple depths and proposes a new ocean-temperature time-series prediction method based on the temporal dependence parameter matrix fusion of historical observation data. The Temporal Dependence-Based Long Short-Term Memory (LSTM) Networks for Marine Temperature Prediction (TD-LSTM) proves better than other methods while predicting sea-surface temperature (SST) by using Argo data. The performances were good at various depths and different regions.

## 1. Introduction

Seawater temperature is an important indicator that measures water heat and is, thus, one of the most important physical factors of the marine environment. The changes in seawater temperature depend on the process of solar radiation, heat exchange between atmosphere and seawater, evaporation, submarine earth activity, ocean internal radioactive fission and a biochemistry process [1]. Oceans cover three-quarters of the Earth and their specific heat capacity is rather high, with the ocean playing an important role in the regulation of the global climate. Ocean-temperature prediction makes humans better understand global climate change and marine ecosystems. There are many factors that can result in the change of water temperature, mainly divided into the influence of time and space [2]. Spatiality is embodied in different areas that have different seawater temperature. The farther the distance, the more obvious the temperature differences are. Timeliness is reflected in water temperature in different times, different quarters, and different years are various. Due to ocean temperature being affected by many factors, complex and changeable, carrying on an accurate prediction of space and time at the same time is a challenge [3].

Ocean-observing networks are essential for describing and understanding the ocean [4]. For the purpose of better observing the ocean and knowing more about the ocean, many large-scale global ocean-observation programs have been in progress, including the World’s Oceans Real-Time Network Plan (ARGO), the Integrated Ocean Observation System (IOOS), the "NEPTUNE" seafloor observatory network planning (NEPTUNE), and European programs such as ESONET and DONET. These observed data have been used for numerical results comparison and also to increase knowledge on global ocean and climate [5]. Moreover, ocean observed data prediction depends on observed arrays and tools that are able to estimate forecasts [6].

In general, ocean forecasting methods are mainly divided into two categories: numerical-calculation and empirical-statistics methods. The numerical-calculation method is to establish a prediction model through kinetic and thermal equations, and make a numerical solution after given numerical conditions and boundary conditions [7]. From traditional empirical statistics methods to artificial intelligence approaches, there are many data-driven techniques for predicting ocean temperatures [8]. The representative statistical techniques include Markov model [9], empirical canonical correlation analysis [10]. However, in recent years, the neural network has been used successfully in many applications.

In marine-temperature research, the neural network plays a very important role. In [11], Tangang et al. used neural-network models to seasonally forecast tropical Pacific sea-surface temperature anomalies (SSTA) in the Niño 3.4 region (6${}^{\circ }$ S–6${}^{\circ }$ N, 120${}^{\circ }$ W–170${}^{\circ }$ W). Wu et al. predicted the SST over the tropical Pacific using a neural network (NN) that consisted of deriving the SST principal components over a three- to 15-month lead time using the input of SST and sea level pressure [12]. In [13], Tripathi et al. analyzed the SST data in the Indian Ocean and used the ANN technique to study the predictability of the Indian summer monsoon. It has been found that the performance of the ANN model was better than the corresponding regression model in the prediction of Indian summer-monsoon rainfall.

Marine Temperature has the characteristic of changing with time, which means that each point contains a large amount of data. Recurrent NNs (RNN), as an important form of neural network, is used to process sequence data. However, RNN suffers a lot from vanishing- or exploding-gradient problems that cannot solve the long-term dependence problem. As a special RNN, Long short-term memory (LSTM) introduces the gate mechanism and can prevent back-propagated errors from vanishing or exploding [14]. In general, LSTM performs better than RNN and the Hidden Markov Model (HMM) [15]. Zhang et al. adopted LSTM in their research to predict sea-surface temperature (SST) and made a short-term prediction, including one day and three days, and long-term prediction, including weekly mean and monthly, mean [16]. Yang et al. proposed a model that combines temporal and spatial information to predict future SST values. Its structure includes one fully connected LSTM layer and one convolution layer [17].

Marine data have the characteristics of wide distribution, a large amount of data, and many types. Effective use of cloud-computing resources benefit the development of large-scale high-performance-computing ocean data. Fustes et al. proposed a new segmentation method to isolate dark areas in SAR images. Cloud computing is used for scaling up the algorithms and providing communication between users [18]. The above research shows how their approach benefits from cloud resources.

In this article, we put forward a TD-LSTM network to predict ocean-temperature changes. In TD-LSTM, we first analyze the temperature history of marine observation values, refer to their closeness, period, and trend, using the ocean-temperature fusion method based on time dependence to reconstruct the data sequence. According to the reconstructed LSTM model, we can predict the time series of ocean temperatures.

This paper’s main contributions include: (1) by analyzing the time dependence of ocean-temperature changes, the ocean-temperature fusion method is proposed based on time dependence; (2) putting forward an LSTM ocean-temperature prediction method based on time dependence; (3) extension based on a time-dependent LSTM ocean-temperature prediction method is applied to different depths; (4) verifying the validity of the TD-LSTM in different areas.

The rest of this paper is organized as follows. In Section 2, we describe the problem formulation and long short-term memory networks in our paper. Section 3 puts forward an analysis method about temporal dependence and a method to construct TD-LSTM. Simulation results and performance evaluations are shown in Section 4. Section 5 presents our conclusions.

## 2. Preliminary

### 2.1. Problem Formulation

The discrete time sequence is a set of chronological observation values, temperature, salinity, dissolved oxygen, and daily precipitation are all sequences of this kind.

**Figure 1:**
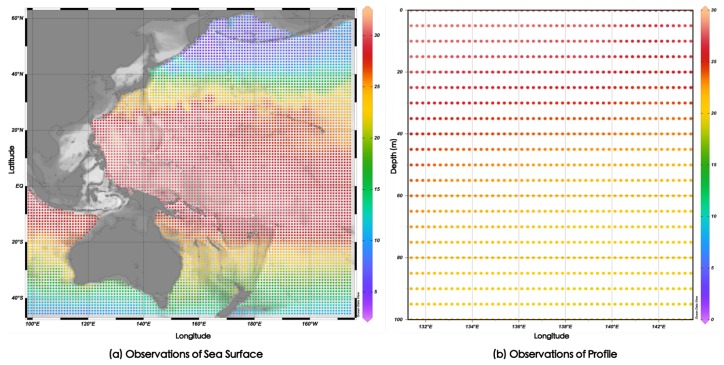
Grid data of ocean temperature.

The 3D grid data sets consist of longitude, latitude, and depth. As shown in Figure 1a, in the horizontal plane, each observation point has corresponding observed values. At the same time, as shown in Figure 1b, in each observation point in the vertical direction of different depth also has the corresponding measurements. These points make up the spatial distribution of ocean temperature. On time distribution, each temporal point has a set of observations sorted by time interval. We wanted to solve the problem of how to predict future data according to existing temperature data.

We look at the observations at each observation point as a time series. If a model can be built to capture the temporal dependencies among data, then the future values can be predicted according to the historical values. Therefore, the prediction problem of each observation point in the 3D grid region can be expressed as the prediction problem of time series. Given the historical observations $\left\{X_{t}|t=0,\ldots ,n-1\right\}$, predict $\left\{X_{n}\right\}$.

### 2.2. Long Short-Term Memory

An LSTM network is a special form of RNN, which is a type of gated RNN. LSTM was proposed by Hochreiter and Schmidhuber in 1997. In the earliest LSTM model, the ingenious concept of self-circulation was introduced to generate a path for the long-term continuous flow of gradients [19]. One of the most important extensions was to make the weight of self-looping context-sensitive rather than fixed. This overcomes, to some extent, the most direct gradient disappearance or explosion problem caused by a traditional RNN due to an excessive number of layers in the time dimension. By using the hidden layer as a memory unit, the LSTM network can handle correlations between time series, both for short and long periods of time. Among them, the gated structure controls the weight of the self-loop, and the accumulated time scale can be dynamically changed. Even for the LSTM of fixed parameters, the time scale can be changed due to the input sequence.

**Figure 2:**
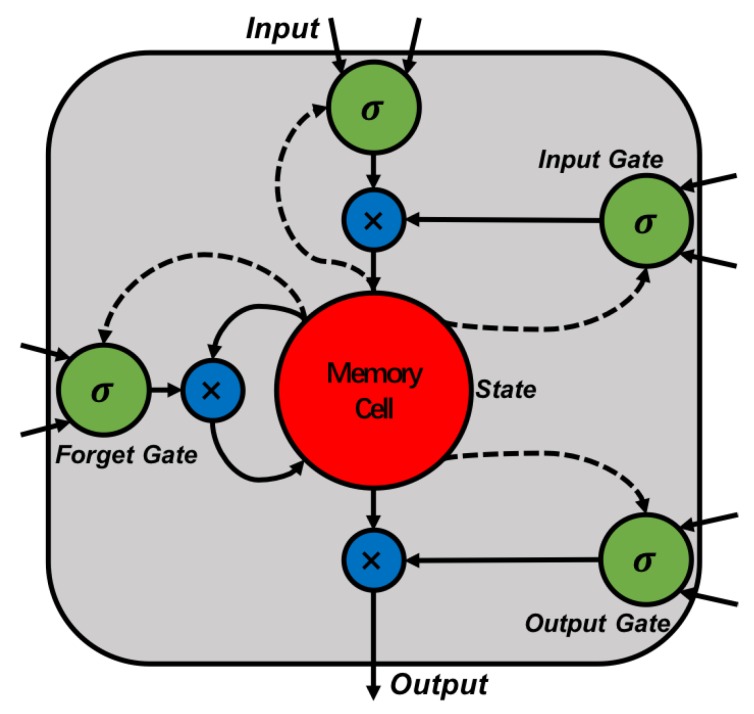
Structure of the long short-term memory (LSTM) cell.

The LSTM model actually uses an LSTM cell structure to replace the cellular structure of the RNN hidden layer, giving it the ability to remember for long periods of time. After continuous improvement, the cell structure of the most widely used LSTM model is shown in Figure 2. Among them, the input gate controls whether its value can be added to the state (“memory cell”). The state unit can be linearly self-looping, and the forgetting gate controls its weight. The output of the cell is controlled by an output gate that can be controlled to turn off. All gating units can perform a sigmoid nonlinear transformation, and the input unit can have any compression nonlinearity. The entire calculation of the LSTM network can be defined by a series of equations as follows:

(1) $$i_{t}=\sigma \left(W^{i}H+b^{i}\right)$$

(2) $$f_{t}=\sigma \left(W^{f}H+b^{f}\right)$$

(3) $$o_{t}=\sigma \left(W^{o}H+b^{o}\right)$$

(4) $$c_{t}=\tanh\left(W^{c}H+b^{c}\right)$$

(5) $$m_{t}=f_{t}⨀m_{t-1}+i_{t}⨀c_{t}$$

(6) $$h_{t}=\tanh\left(o_{t}⨀m_{t}\right)$$

Among the equations, $i_{t}$, $f_{t}$, $o_{t}$, $c_{t}$ denote enter threshold values, forgetting gate values, output gate values, and new states of memory cells. σ is a sigmoid function, and $W^{i}$, $W^{f}$, $W^{o}$, and $W^{c}$ are weight matrices. $b^{i}$, $b^{f}$, $b^{o}$, and $b^{c}$ are the corresponding offset terms. *H* is the concatenation of the new input $x_{t}$ and the previous hidden vector $h_{t-1}$. $m_{t}$ is the final state of the memory cell, and $h_{t}$ is the final output of the memory unit.

## 3. Methodology

The temporal dependence-based LSTM (TD-LSTM) network is mainly divided into two parts. The first part is to analyze the temporal dependence of marine-temperature changes and propose a temporal dependence parameter matrix fusion method. The second part is to use the fusion sequence to train the LSTM neural network to obtain the prediction model.

### 3.1. Temporal Dependence Analysis

LSTM networks have the ability to learn the long-term temporal dependence of marine temperature. However, the desire to simulate the periodicity and trend of temperature changes requires a very long input sequence. This will lead to a lengthy process throughout the training process. In order to ensure the accuracy of the prediction while reducing the amount of training, we will use the ocean temperature time closeness, trend and period to select primary data for modeling.

**Figure 3:**
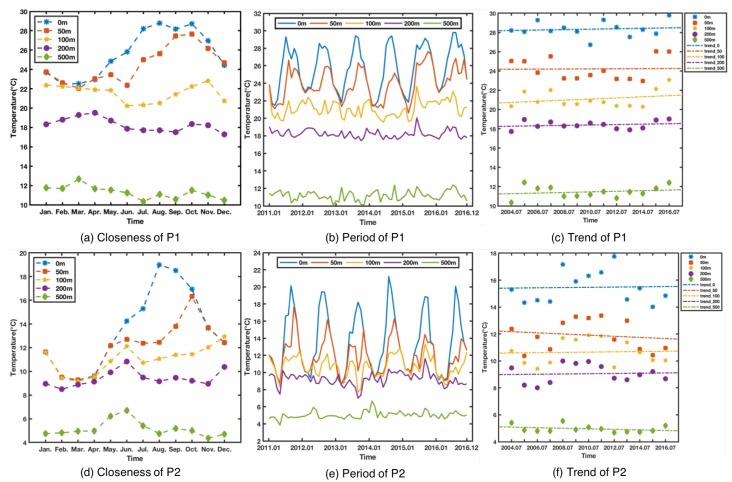
Temporal dependencies in different observations.

Figure 3 shows temporal dependencies in different observations. Figure 3a,d show temperature profiles at two different depth in the ocean. The *X* axis represents the time interval (from January to December) and the *Y* axis represents the monthly average temperature of the corresponding month. The curves from different depths all show an empirical temporal correlation in time series, i.e., the temperature of recent time intervals are more relevant than ones of distant time intervals. This indicates that the ocean temperature has closeness in the time dimension. The five curves have different shapes, indicating that different closeness at different depths. At the same time, comparing Figure 3a,d from different latitude and longitude, it shows that different regions also have different characteristics of closeness. In Figure 3b,e, we can clearly observe that the annual period. The period distribution is very clear at 0 and 50 m, but not so obvious at 100, 200, and 500 m. Figure 3c,f show the trend of monthly mean temperature for July at different depths during the period of 2004–2016. It can be seen that the trend of temperature’s increase or decrease is different at different depths. Temperature changes at different locations and depths also various. In general, their temperature changes are affected by their closeness, period and trend, but the degree of impact may be different.

Trends are usually reflected by observations, where we use multiple periodic points to represent trends. Inspired by the above analysis, observations at different depths are affected by closeness, period, and trend. We propose a temporal dependence parametric matrix fusion method, as shown below.

(7) $$X_{TD}=W_{c}\circ X_{c}+W_{p}\circ X_{p}$$

Among the quotations, ∘ is Hadamard product, $X_{c}$ and $X_{p}$ represent closeness and period, respectively, $W_{c}$ and $W_{p}$ are the parameters used to weight the closeness and period. $W_{c}\circ X_{c}$ is the length of closeness sequence and $W_{p}\circ X_{p}$ make the lehgth of period sequence. $W_{c}\circ X_{c}$ and $W_{p}\circ X_{p}$ together decide the length of trend. For a single observation, it will become a temporal sequdence after fusion [20].

### 3.2. Structure of TD-LSTM Networks

**Figure 4:**
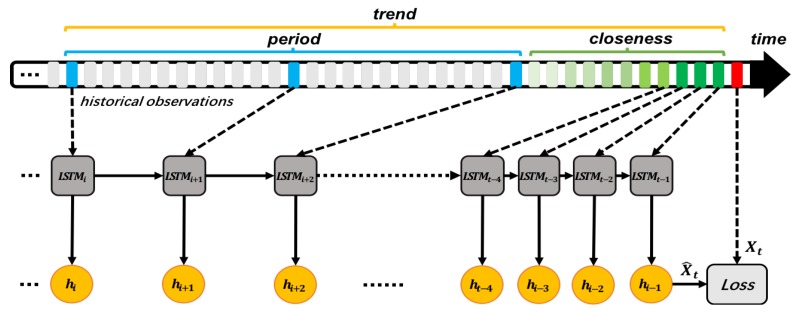
TD-LSTM networks architecture.

**Figure 5:**
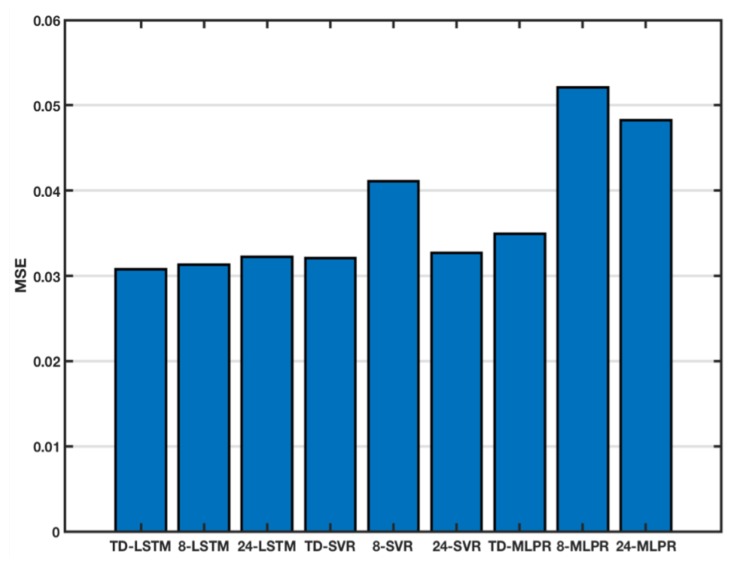
The average of MSE.

In the previous section, we discussed how to choose keyframes for the model. This reduces training time and capture time-dependent features. Figure 4 shows the structure of our proposed TD-LSTM networks. As illustrated in the top part of Figure 4, there is a set of historical observations of ocean temperature. The red dot indicates the temperature at which time t is to be predicted. We used green dots to reflect the closeness of time. As time distance increases, the relationship between temporal closeness becomes weaker and green becomes shallower. We used blue dots to reflect the period of time, they have the same time interval. The closeness and periodic points together reflect the trend. We combined these points that reflect closeness, period, and trend, and put them in sequentially into the LSTM units in the middle of Figure 5. The lower part of Figure 5 shows the output value corresponding to each LSTM unit state. When the input at time t−1 is completed, the LSTM network gives the predicted value at time *t*. The predicted value is compared with the real value, and the Loss value is calculated to optimize the network.

### 3.3. Algorithm and Optimization

In the following part, Algorithm 1 is presented for the training TD-LSTM networks at an observation point. We first constructed the training instances from the original observations. Then, TD-LSTM was trained via back-propagation [21] and optimized by Adaptive Moment Estimation (Adam). Adam is an adaptive-learning rate-optimization algorithm. Its advantage mainly lies in that, after bigotry correction, the learning rate of each iteration has a certain range, which makes the parameters relatively stable [22].

**Algorithm 1** Training of TD-LSTM networks
**Input:**
 historical observations:$\left\{X_{1},\ldots ,X_{n-1}\right\}$; target at time *t*: $X_{t}$; lengths of closeness, period, trend: $l_{c},l_{p}$; period: *p*;
**Output:**
 TD-LSTM model M; //construct training instance 1: D←⌀; 2: **for** all available time interval t(1≤t≤n−1)
**do** 3: $S_{c}=\left[X_{t-l_{c}},X_{t-(l_{c}-1)},\ldots ,X_{t-1}\right]$; 4: $S_{p}=\left[X_{t-l_{p}\cdot p},X_{t-(l_{p}-1)\cdot p},\ldots ,X_{t-p}\right]$; 5: put a training instance ($\left\{S_{c},S_{p}\right\}$) into D; 6: **end for** // train model 7: initialize parameter θ; 8: **repeat** 9: randomly select a batch of instances from D;10:   find θ by optimization algorithm;11: **until** the objective is minimized12: output the optimized TD-LSTM model M;

## 4. Experiment

In this section, we evaluated our method on BOA_Argo against a few methods.

### 4.1. Settings

#### 4.1.1. Datasets

The data used in this experiment is from the Global Ocean Argo Grid Data Set (BOA_Argo) [23]. The grid dataset provides annual average, monthly average and yearly ocean temperature from January 2004 to December 2017 covering the global ocean (180${}^{\circ }$ W 180${}^{\circ }$ E, 79.5${}^{\circ }$ S 79.5${}^{\circ }$ N) and salinity data. Spatial resolution is 1${}^{\circ }$ × 1${}^{\circ }$ horizontally and 58 layers in the vertical standard layer.

#### 4.1.2. Baselines

We compare our TD-LSTM with the following three baselines:

1. SVR
  Support Vector Regression (SVR) is one of the most popular regression models in recent years. SVR has achieved good results in many applications. In the experiment, we used support vector regression with the RBF kernel function.
2. MLPR
  Multilayer Perceptron Regressor (MLPR) is a typical artificial neural network for regression tasks. We used a three-layer perceptron network, which includes one hidden layer with 100 neurons. In the MLPR network, we set activation= ${}^{\prime }relu^{\prime }$ and α=0.0001.
3. LSTM
  LSTM is a time-recurrent neural network. Thanks to its unique design structure, LSTM can learn long-term correlation. In the experiment, we used 8 and 24 m LSTM to compare with our proposed method.

In the experiment, we used different variants of the above methods as needed.

#### 4.1.3. Preprocessing

We use the Min-Max normalization method to scale the time series data into the range [−1, 1]. In the evaluation, we got the predicted value back to normal values with inverse transform.

#### 4.1.4. Hyperparameters

In the TD-LSTM networks, the parameters that can be learned using the default parameters provided by Keras. In the LSTM layer, we used the tanh activation function. It is a fraction of the units to drop for the linear transformation of the inputs. We used Adam as the gradient optimization algorithm, as Adam is an adaptive learning rate optimization algorithm [22]. Adam is generally pursuing as being fairly robust to The choice of hyperparameters, although the learning rate sometimes needs to be changed from the suggested default [24]. When we are dependent on the sequence length, we compare the different $l_{c}$, and $l_{p}$ length combinations. In the allocation of training and validation sets, we randomly selected 90 percents of the training data to train each model and then used the remaining 10 percents of the data as the validation set. Finally, we train the final model using all the training data.

#### 4.1.5. Evaluation Metric

We measure our method by Mean Squared Error (MSE),

(8) $$MSE=\frac{SSE}{n}=\frac{1}{n}\sum_{i=1}^{n}{\left(y_{i}-\hat{y_{i}}\right)}^{2}$$

Here, there are *n* samples, $\hat{y_{i}}$ is the predicted value of the *i*th sample, and $y_{i}$ is the corresponding true value. The smaller the MSE, the more accurate the description of the experimental data is proved by the prediction model.

### 4.2. Evaluation of SST Prediction

In this section, we first evaluated the predictive performance of TD-LSTM for sea surface temperatures. Taking the world’s largest sea-coral sea area as an example, five observation points were randomly selected, a time-dependent parameter matrix fusion was performed on their historical observation data, and the TD-LSTM model was trained using the fused sequence to predict the temperature of the next month. We set the time-dependent parameter of TD-LSTM to $l_{c}=6$, $l{}_{p}=2$. For better comparison, other methods have the same input sequence as TD-LSTM. We also set different input sequence lengths for comparison, which were 8 (months) and 24 (months), respectively. Since 8 has the same sequence length as $l_{c}=6$, $l_{p}=2$, 24 has the same time span as $l_{c}=6$ and $l_{p}=2$. Table 1 shows the MSE of all methods on BOA_Argo at SST of Coral Sea. The results show that TD-LSTM performance is not the most prominent at each point, but the average MSE is the best. In other words, TD-LSTM is better than other methods in overall forecasting. Figure 5 shows the average MSE for each model at 5 locations. It can be seen that TD-LSTM has the best performance. TD-LSTM is 1.6% better than 8-LSTM, 4.3% better than 24-LSTM, 4% better than TD-SVR, 25.1% better than 8-SVR, 6.2% better than 24-SVR, and 11.7% better than TD-MLPR. It is 40.9% better than 8-MLPR and 36.1% better than 24-MLPR.

### 4.3. Results of Different TD-LSTM Variants

In the previous section, we compared the performance of the TD-LSTM method in predicting SST. In this section, we extend the method to different depths and regions to demonstrate the possibilities of TD-LSTM for more applications.

#### 4.3.1. Comparisons of Different Ocean Depth

**Figure 6:**
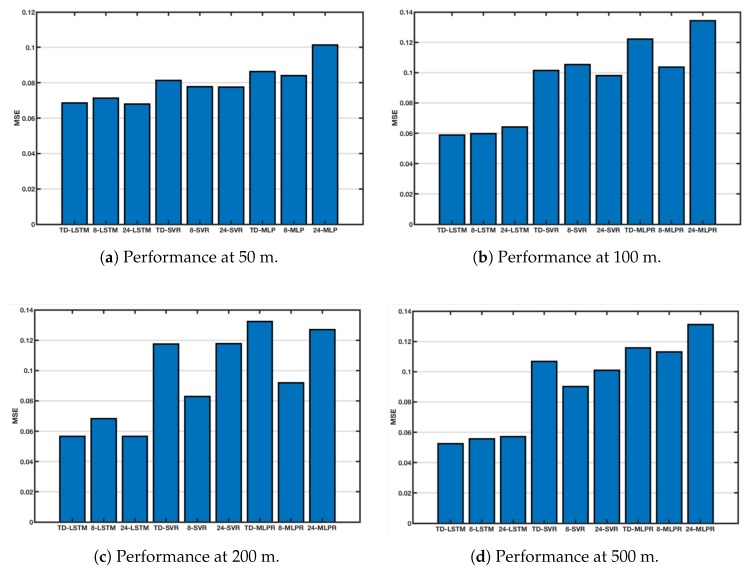
Comparison of performance at different depths.

As shown in Figure 3, seawater of different depths has different variations. The four figures in Figure 6 show the performance of each method at 50 m, 100 m, 200 m, and 500 m. MSE is still the average MSE of 5 points randomly selected. Figure 6a compares the performance at 50 m, and the performance of TD-LSTM ranks second, without the 24-LSTM. This is because the input sequence used by TD-LSTM is only 1/3 of the length of 24-LSTM. With a reduced number of input sequences, performance is close to 24-LSTM and can be tolerated. The performance of TD-LSTM is better than other variants of SVR and MLP. Figure 6b compares performance at 100 m depth. TD-LSTM performs better than 24-LSTM, much better than SVR and MLP variants. The performance of TD-LSTM is better than that of 24-LSTM because the variation of seawater temperature at 100 m depth is not obvious. The time-dependent parameter matrix fusion method can better grasp the characteristics of the variation law. Figure 6c,d compare the performance of each method at 200 m and 500 m, respectively. The LSTM method is better than the variants of SVR and MLPR. The performance of the TD-LSTM is close to that of the 24-LSTM and is better than the 8-LSTM.

#### 4.3.2. Impact of Temporal Closeness, Period and Trend

**Figure 7:**
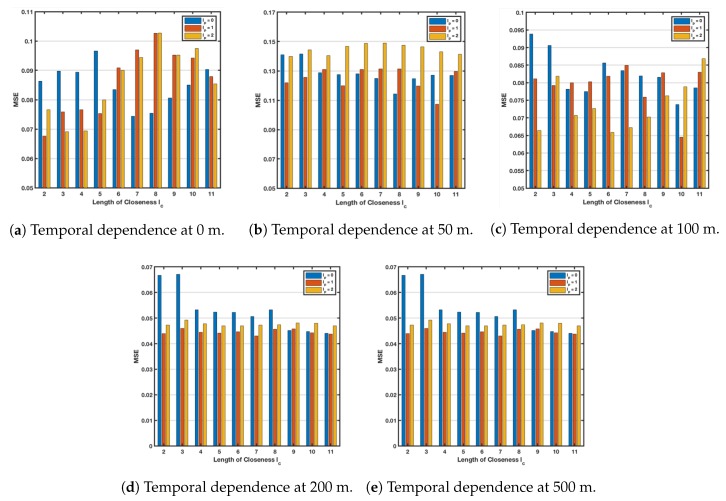
Impact of temporal dependences at different depths.

To prove the effect of time dependence on the performance of the model, we choose the closeness length $l_{c}$ from 2 to 11 and the periodic length $l_{p}$ from 0 to 2 to compare the performance of their various combinations. The results at depths of 0 m, 50 m, 100 m, 200 m and 500 m are shown in Figure 5, respectively. We use the x-axis to indicate the number of closeness lengths, three colors to indicate the number of periodic lengths from 0 to 2, and the y-axis to the corresponding MSE value. Figure 7a shows the model performance of each parameter combination on the sea surface (0 m). By comparison, at a depth of 0 m, $l_{c}=2$, $l_{p}=1$, the model obtains the best MSE. As shown in Figure 7b, the best performance of the model obtained when $l_{c}=10$, $l_{p}=1$. Figure 7c reflects the results at 100 m, $l_{c}=10$, $l_{p}=1$ bring the best performance. At a depth of 200 m, $l_{c}=7$, $l_{p}=1$, the model obtained the best MSE. In the case of a depth of 500 m, $l_{c}=4$, $l_{p}=1$ was the best selection.

With the same comparison method, the choices obtained at different depths are different. This result tells us that at different depths, time-dependent parametric matrix fusion should be performed for different depths and selecting appropriate parameters. Only in this way can we get a better model.

### 4.4. Comparisons of Different Regions

**Figure 8:**
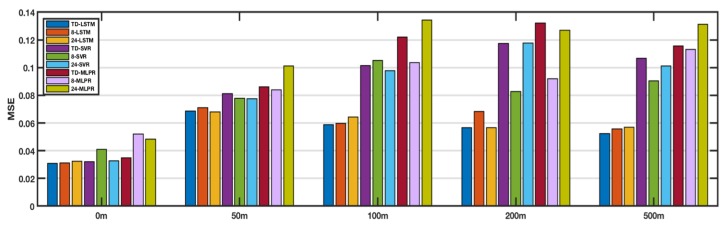
Comparison of performances at different depth in the Coral Seas.

**Figure 9:**
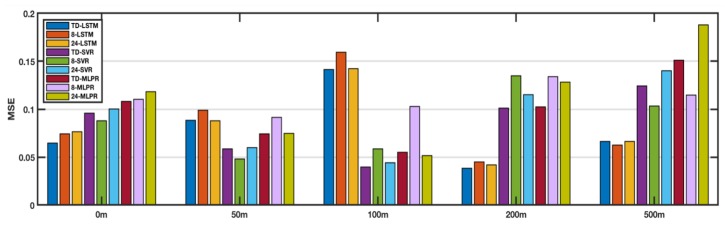
Comparison of performances at different depth in the Equatorial Pacific Region.

**Figure 10:**
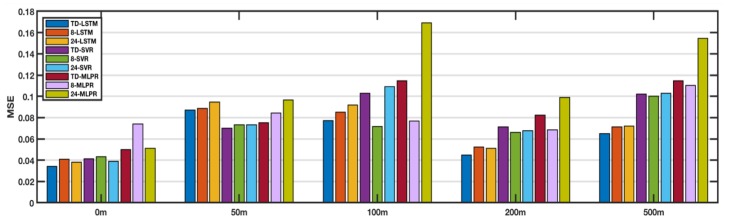
Comparison of performances at different depth in the South China Sea.

In this section, we applied the TD-LSTM method to different regions, including the Coral Sea, the Equatorial Pacific Region, and the South China Sea. The performance of the method was evaluated by comparing the average of MSEs of a plurality of randomly selected points. Figure 8 shows the comparison of various methods in the Coral Sea. The results show that LSTM is better than SVR and MLPR. Between LSTM variants, TD-LSTM is better than 8-LSTM and 24-LSTM, and TD-LSTM has an input sequence length less than 24-LSTM, which reduces the need for historical-data collection and reduces the computational overhead of the model. Figure 9 shows the comparison of the various methods in the Equatorial Pacific Region. The results show that the LSTM method is better than SVR and MLPR at 0 m, 200 m, and 500 m. Among the variants of LSTM, TD-LSTM is better than 8-LSTM and 24-LSTM. At 50 m and 100 m, LSTM is not as good as SVR. Figure 10 shows the comparison of the various methods in the South China Sea. The results are similar to those in the Pacific equatorial region. At 0 m, 200 m, and 500 m, the LSTM method is better than SVR and MLPR. Among the variants of LSTM, TD-LSTM performs best. At 50 m and 100 m, SVR performs better than LSTM.

Combining the results of the above three sea areas, it is not difficult to find that LSTM has better performance than SVR and MLPR at 0, 200, and 500 m. In the variant of LSTM, TD-LSTM performs better thasn 8-LSTM and 24-LSTM. Table 2 shows the improvement rate of TD-LSTM relative to other methods at 0, 200, and 500 m. It can be seen from the numbers that the overall performance of TD-LSTM at three depths is the best, far better than the variants of SVR and MLPR.

## 5. Conclusions

In this paper, we analyzed the temporal dependence (closeness, period, and trend) of marine temperature, and proposed a method based on temporal dependence parameter matrix fusion for historical observation ocean temperature data. Using the fusion data, the LSTM network, a time-dependent LSTM ocean temperature prediction model is trained and the method is proposed. We evaluated our model using BOA_Argo data. Experiments have shown that the TD-LSTM and its variants are better than SVR and MLPR in multiple seas (the Coral Sea, the Equatorial Pacific Region, and the South China Sea). The input sequence length that we used to train the model can be reduced by using the TD-LSTM method. In terms of prediction accuracy, the performance of TD-LSTM is close to or even better than those methods, whose time span are similar to TD-LSTM, but the input sequence length is much longer than it. The superior performance of the TD-LSTM was demonstrated at multiple depths in multiple sea areas.

## Figures and Tables

**Table 1:** Comparison of SST predictions in the coral sea area.

Methods	P1	P2	P3	P4	P5	Average of MSE
TD-LSTM	0.0525	0.0319	0.0158	0.0305	0.0232	0.0308
8-LSTM	0.0576	0.0293	0.0129	0.0287	0.0280	0.0313
24-LSTM	0.0611	0.0314	0.0162	0.0297	0.0227	0.0322
TD-SVR	0.0368	0.0269	0.0296	0.0321	0.0348	0.0321
8-SVR	0.0414	0.0500	0.0203	0.0536	0.0403	0.0411
24-SVR	0.0389	0.0229	0.0294	0.0455	0.0267	0.0327
TD-MLPR	0.0361	0.0316	0.0353	0.0428	0.0288	0.0349
8-MLPR	0.0622	0.0580	0.0302	0.0689	0.0411	0.0521
24-MLPR	0.0617	0.0282	0.0503	0.0715	0.0295	0.0482

**Table 2:** Comparison of TD-LSTM performance improvement rates.

Region	Coral Sea			Equatorial Pacific Region			South China Sea		
Methods	0 m	200 m	500 m	0 m	200 m	500 m	0 m	200 m	500 m
8-LSTM	1.7%	17%	5.7%	13.2%	14.2%	−6.0%	16.0%	13.9%	8.8%
24-LSTM	4.6%	−0.4%	7.9%	15.5%	8.3%	0.03%	10.3%	11.9%	10.1%
TD-SVR	4%	51.7%	50.8%	32.4%	62.1%	46.5%	7.2%	36.8%	36.4%
8-SVR	25.2%	31.5%	41.9%	26.4%	71.6%	35.8%	20.3%	31.8%	35.2%
24-SVR	5.9%	51.8%	48.1%	35.4%	66.7%	52.6%	3.9%	33.7%	37.1%
TD-MLPR	11.9%	57.1%	54.6%	40.3%	62.5%	56.1%	31.4%	45.4%	43.4%
8-MLPR	40.9%	38.3%	53.6%	41.4%	71.3%	42.1%	53.7%	34.5%	41.2%
24-MLPR	36.2%	55.4%	60%	45.2%	70.0%	64.6%	32.9%	54.5%	58.0%
